# Molecular analysis of high-grade serous ovarian carcinoma with and without associated serous tubal intra-epithelial carcinoma

**DOI:** 10.1038/s41467-017-01217-9

**Published:** 2017-10-17

**Authors:** Jennifer Ducie, Fanny Dao, Michael Considine, Narciso Olvera, Patricia A. Shaw, Robert J. Kurman, Ie-Ming Shih, Robert A. Soslow, Leslie Cope, Douglas A. Levine

**Affiliations:** 1 Department of Surgery, Memorial Sloan Kettering Cancer Center, New York, NY 10065 USA; 2 Sidney Kimmel Comprehensive Cancer Center at Johns Hopkins, Baltimore, MD 21287 USA; 3 Department of Pathology, University Health Network, Toronto, Canada M5G 2C4; 4 Departments of Pathology, Gynecology and Obstetrics and Oncology, Johns Hopkins Medical Institutions, Baltimore, MD 21205 USA; 5 Department of Pathology, Memorial Sloan Kettering Cancer Center, New York, NY 10065 USA; 6 Present Address: Gynecologic Oncology, Mercy Medical Center, Baltimore, MD 21202 USA; 7 Present Address: Laura and Isaac Perlmutter Cancer Center, New York University Langone Health, New York, NY 10016 USA

## Abstract

Many high-grade serous carcinomas (HGSCs) of the pelvis are thought to originate in the distal portion of the fallopian tube. Serous tubal intra-epithelial carcinoma (STIC) lesions are the putative precursor to HGSC and identifiable in ~ 50% of advanced stage cases. To better understand the molecular etiology of HGSCs, we report a multi-center integrated genomic analysis of advanced stage tumors with and without STIC lesions and normal tissues. The most significant focal DNA SCNAs were shared between cases with and without STIC lesions. The RNA sequence and the miRNA data did not identify any clear separation between cases with and without STIC lesions. HGSCs had molecular profiles more similar to normal fallopian tube epithelium than ovarian surface epithelium or peritoneum. The data suggest that the molecular features of HGSCs with and without associated STIC lesions are mostly shared, indicating a common biologic origin, likely to be the distal fallopian tube among all cases.

## Abstract (web summary)

High-grade serous carcinomas (HGSCs) are associated with precursor lesions (STICs) in the fallopian epithelium in only half of the cases. Here the authors report the molecular analysis of HGSCs with and without associated STICs and show similar profiles supporting a common origin for all HGSCs.

## Introduction

Ovarian cancer has traditionally been thought to develop from the ovarian surface epithelium (OSE) or cortical inclusion cysts (CICs), but the recent data suggest that a majority of advanced high-grade serous carcinomas (HGSC) likely originate from the epithelium of the distal fallopian tube^1^. Serous tubal intra-epithelial carcinoma (STIC) lesions are suspected to be the precursor for most HGSCs of the pelvis. STIC lesions have been demonstrated to have a clonal relationship with established HGSCs based on both shared *TP53* mutations as well as integrated molecular analyses^2–4^. Originally identified in the fimbriated end of serially sectioned fallopian tubes, prophylactically removed from women at high-risk for developing ovarian cancer, these lesions have since transformed our understanding of the origins of this malignancy. It is now clear that with detailed processing of the tubal fimbria, many cases of presumed ovarian cancer contain evidence of tubal origin as manifested by the presence of a STIC. For example, recent reports have documented that 40–60% of advanced pelvic HGSCs contain identifiable STICs^4–7^. The absence of STIC lesions in a higher proportion of HGSCs may be due to tubal overgrowth and STIC obliteration by the invasive tumor, lesions not sampled through contemporary pathology protocols, or shedding of normal tubal cells prior to malignant transformation.

Alternatives to the tubal hypothesis suggest that the tubal epithelium is colonized in a metastatic manner from the primary ovarian tumor or that some pelvic HGSCs originate from tubal epithelium and others originate from other pelvic structures such as the “secondary Müllerian system”^8^. The former has been disproven using studies of telomere length and clonality^9^. The latter is biologically plausible, but with little concrete data for support. If either of these alternative hypotheses were correct, we would then expect to find molecular differences between HGSCs with and without associated STICs.

Molecular profiling has yielded promising results in the field of predictive classifiers, led to a greater understanding of the pathobiology of cancer, and moved us toward precision medicine. Robust examples can be found across many solid tumors, including breast, colon, ovarian, and endometrial cancer. In ovarian cancer, studies have defined molecular subtypes of advanced ovarian carcinoma, identified deregulated cancer-related biologic pathways, and identified therapeutically relevant predictive classifiers. In a limited number of cases, these models are sufficiently reliable and robust to consider direct application in clinical practice^10–12^.

Although highly provocative, these hypotheses require further validation to determine whether there are subsets of HGSC, which have different molecular profiles based on their presumed site of origin or whether there are no differences, and they are essentially indistinguishable based on putative sites of origin. A multi-institutional analysis of pelvic HGSC with STICs uniformly classified will provide a unique assessment of the molecular profiles to determine if a uniform tissue of origin exists. We have analyzed a large set of tubo-ovarian HGSCs diagnosed using traditional criteria with and without STICs in women whose fallopian tubes have been processed using the SEE-FIM technique, currently the most comprehensive method of evaluating fallopian tube epithelium. We tested the hypothesis that advanced pelvic HGSCs with or without associated STICs have similar clinical characteristics, indicating a common underlying origin from the fallopian tube. As described in detail in “Methods” section, we designed the study with sufficient power to detect most molecular differences in the moderate to large range, reasoning that a negative result would provide strong evidence in support of the single origin theory.

## Results

### Description of patient and tumor characteristics

**Fig. 1:**
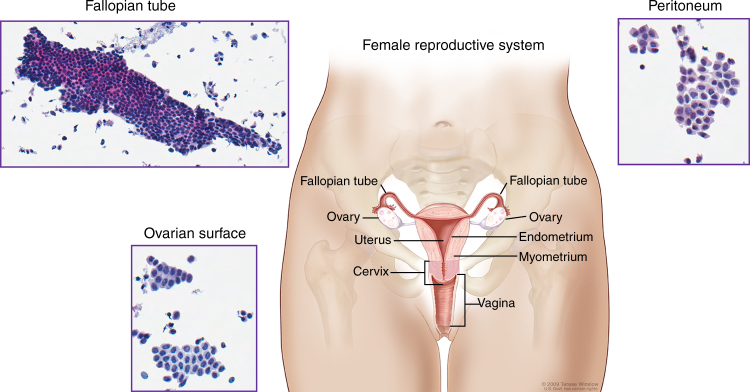
Normal tissue anatomic sites. Pelvic anatomy with insets showing cytologic images of tissue brushings used to obtain pools of normal tissues for molecular profiling. Fallopian tube cytology is predominantly consistent with an epithelial lineage and ovarian surface and peritoneal cytology have mesothelial histology. For the National Cancer Institute^*©*^ (2009) Terese Winslow LLC, U.S. Govt. has certain rights. This image is not included under the creative commons licence for this article

Biospecimens were qualified until an equal number of cases with and without STIC lesions were ascertained to a total of 96 patient samples. The copy number data are available for all 96 samples, as well as the miRNA expression data for 95 samples, and the RNA expression data for 85 samples. The demographic and clinical data are provided in Table 1 and Supplementary Data 1, along with a full accounting of the assays completed for each sample. Briefly, the median age of the study cohort was 59 years. FIGO stage III disease was present in 65% of cases, FIGO stage IV in 29%, and all patients had HGSC. With the exception of formal diagnosis, which changes by definition in the presence of STIC lesions, there were no differences in key clinical features or survival between patients with or without STIC lesions (Table 1 and Supplementary Fig. 1). Normal tissue brushings were representative of epithelial and mesothelial lineages (Fig. 1).

**Table 1:** Demographic and clinical characteristics of patients with high-grade serous carcinomas with and without STIC lesions

	STIC	Non-STIC	Total	*P* Value^b^
Age at diagnosis (years)	59 (41–77)	59 (37–79)		0.4
Median (range)				
Race				0.7
White	36 (75%)	33 (68%)	69	
Black	2 (4%)		2	
Asian	3 (6%)	2 (4%)	5	
Hispanic	1 (2%)		1	
Other	1 (2%)	2 (4%)	3	
FIGO stage				0.5
IA		1 (2%)	1	
II		1 (2%)	1	
IIC	1 (2%)	1 (2%)	2	
III	5 (10%)	9 (19%)	14	
IIIA		1 (2%)	1	
IIIB	1 (2%)		1	
IIIC	24 (52%)	24 (50%)	48	
IV	17 (35%)	11 (23%)	28	
Surgical outcome				0.6
Suboptimal	8 (17%)	5 (10%)	13	
Optimal	39 (81%)	42 (88%)	81	
Platinum sensitivity				0.3
Resistant	8 (16%)	3 (6%)	11	
Sensitive	35 (72%)	32 (67%)	57	
Diagnosis of record				0.07
Fallopian tube	13 (27%)	5 (10%)	18	
Ovarian	29 (60%)	30 (63%)	59	
Primary peritoneal		2 (4%)	2	
Endometrial	1 (2%)		1	
Diagnosis after path review				1e-09
Fallopian tube	40 (83%)	10 (21%)	50	
Ovarian	3 (6%)	25 (52%)	28	
Primary peritoneal		2 (4%)	2	

FIGO International Federation of Gynecology and Obstetrics, Non-STIC no serous tubal intra-epithelial carcinoma, STIC serous tubal intra-epithelial carcinoma

^a^Numbers do not sum to the total of the study population for some variables due to missing values

^b^Fisher’s exact test

### Differential analyses of STIC lesions

**Fig. 2:**
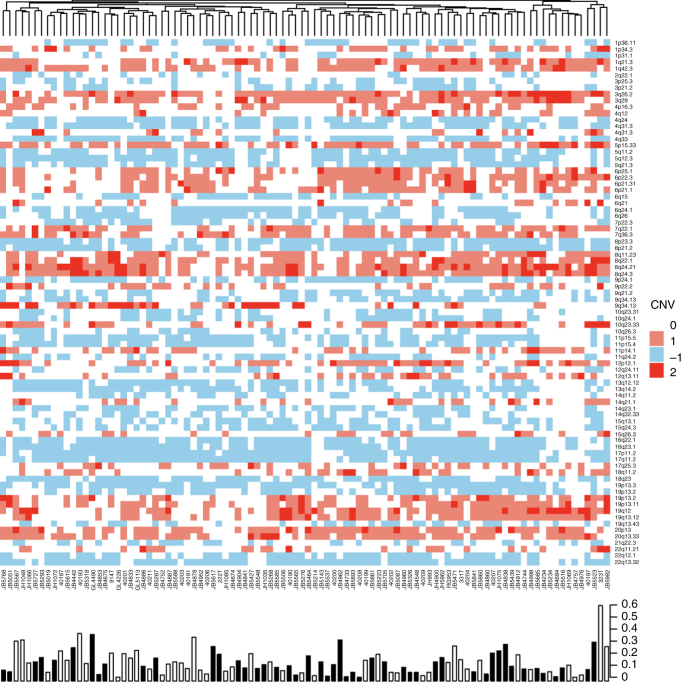
Significant copy number alterations as identified by GISTIC. The proportion of genome significantly altered (homozygous deletion or 2+ fold amplification) is depicted in a bar plot along the bottom. The presence of a STIC lesion is indicated by a black bar in the bar plot

Using the focal somatic copy number alterations (SCNAs) identified from all 96 cases, we compared the frequency distribution between tumors with and without STIC lesions. Of the 82 regions identified as significantly altered in the GISTIC analysis, none were over-represented in either the tumors with and without STIC lesions at a statistically significant level (Supplementary Data 2 and 3). Arm-level changes appear similar between groups when visualized through a heatmap (Fig. 2).

**Fig. 3:**
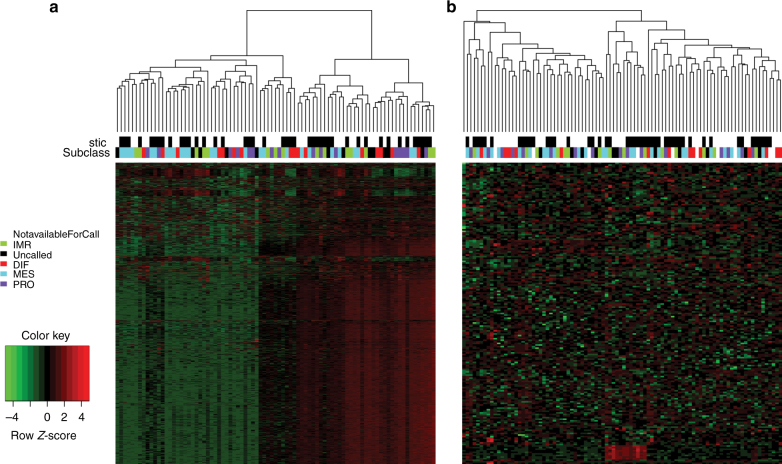
Unsupervised clustering of the most variable genes. Unsupervised clustering analysis of the most variable mRNA (**a**) and microRNA (**b**) transcripts from 85 analyzed tumors with STIC lesion and TCGA subclass indicated in header rows. Samples are in columns and genes in rows

**Fig. 4:**
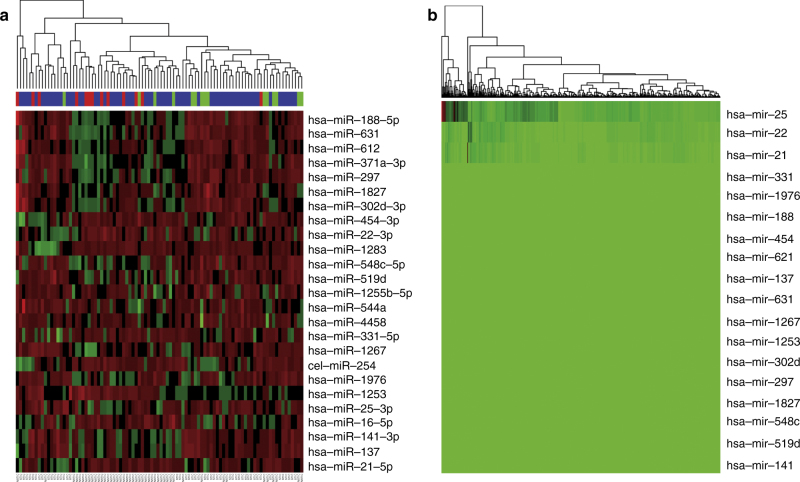
Differentially expressed microRNA. **a** Unsupervised clustering of the 24 microRNAs that were differentially expressed between cases with and without STIC lesions at a corrected *P* value < 0.01. **b** The previously published TCGA data showing that all but three of these genes have low expression based on microRNA sequence abundance

Unsupervised clustering analysis of the most variable mRNA transcripts from 85 analyzed tumors revealed little structure and no clear association with STIC lesions (Fig. 3a). RNAseq analysis was performed in two distinct batches, but the clustering analysis did not show any clear correlation with batch, and STIC and NOSTIC samples were well represented in both groups so no batch correction was performed. Class comparison of the RNA sequencing data identified 199 differentially expressed genes at *P* < 0.01, none of which were statistically significant after multiple comparison correction using the Benjamini–Hochberg step down procedure (Supplementary Data 4). Unsupervised cluster analysis of miRNA data failed to identify a separation between cases with and without STIC lesions (Fig. 3b). Class comparison of microRNA expression identified 24 genes that were differentially expressed between cases with and without STIC lesions at a corrected *P* value < 0.01 (Fig. 4a and Supplementary Data 5). Investigation of these genes within the previously published TCGA data indicates that overall these genes have low expression rendering them likely to be poor candidates for critical roles in ovarian cancer (Fig. 4b). Three microRNAs previously reported to be expressed in various cancer types did have variable and demonstrable expression; mir-22, mir-22, mir-25 (Supplementary Fig. 2).

### Comparison to TCGA HGSC samples

**Fig. 5:**
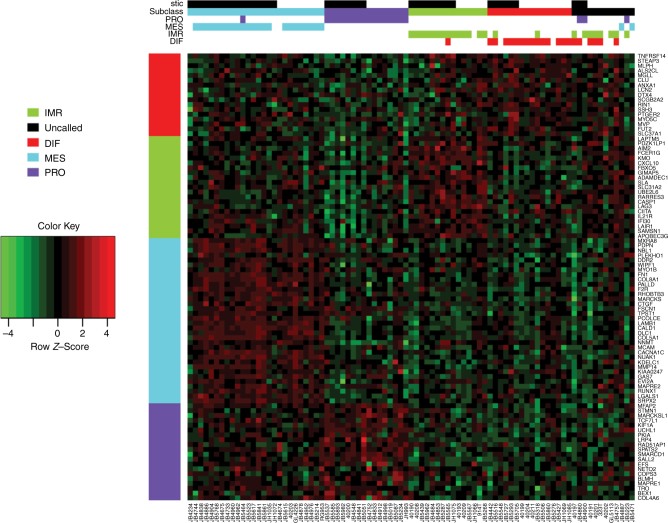
TCGA features of study samples using a gene expression subtype signature. Gene expression using a 100 gene subtype signature and ordered by assigned subtype. Group membership is assigned by color in the top rows for subtypes with > 0.2 correlation. For samples with more than one robust correlation, the highest correlation was assigned if it was at least 1.5 × higher than the next highest subtype correlation. Samples to the far right were not assigned to any particular subtype. STIC status is shown in the top row

**Fig. 6:**
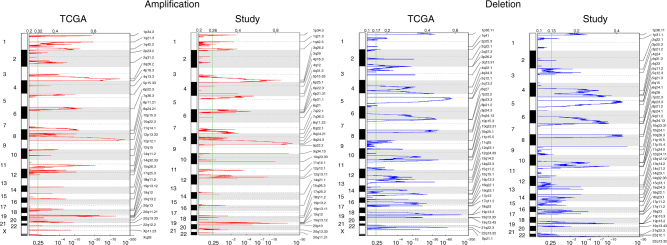
TCGA features of study samples using focal copy number alterations. Focal copy number alterations in study samples compared to the TCGA data

The established gene expression clusters for high-grade serous ovarian carcinoma as previously published were reproduced in the data^12–14^. All four gene expression subtypes were represented in expected proportions. Using a published gene set, subtype membership was assigned when correlations greater than 0.2 were identified (Fig. 5). For samples with more than one robust correlation, the highest correlation was assigned if it was at least 1.5× higher than the next highest subtype correlation. Blocks of overexpression are clearly represented in the samples and correlated to each respective subtype. STIC status does not have a well-defined effect. Among these cases, we did not see a difference in progression-free or overall survival based on gene expression subtype (Supplementary Fig. 3). The most significant focal DNA SCNAs from TCGA were identified in the current study using GISTIC 2.0 (Fig. 6 and Supplementary Data 2 and 3). These regions contained 55% of all focal SCNAs discovered by TCGA for ovarian cancer and 80% of the focal SCNAs that were most significant in TCGA.

### Comparison to normal tissues

**Fig. 7:**
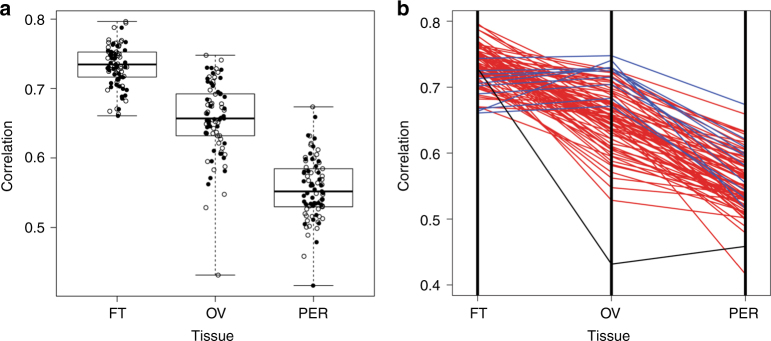
Correlation between gene expression of tumor samples and normal tissues. The mRNA expression profile for each tumor sample was correlated to prototype profiles derived from normal fallopian tube (FT), ovarian surface epithelium (OV) and peritoneal (PER) tissue, respectively, using genes most differentially expressed across normal tissue types. **a** Correlation coefficients for tumor samples compared to each normal tissue type indicated by the presence (solid circles) or absence (open circles) of STIC lesions. Boxplot center lines represent medians, box limits are the inter-quartile range from 25 and 75%, whiskers represent the extent of tumors out to 1.5 times the inter-quartile range. **b** Plot of correlation coefficients for each sample across normal tissue types. Each line corresponds to an individual tumor sample. Seventy-five of eighty-five tumor samples (88%) were most closely correlated with the normal fallopian tube phenotype (red lines). The remaining 10 (blue lines) more closely resembled the normal ovarian tissue. Normal peritoneum tissue had the lowest correlation with all but one sample (black line)

Unsupervised hierarchical clustering of the RNA sequence data from normal tissue pools demonstrated a closer association between OSE and peritoneum, both of known mesothelial lineage, than fallopian tube epithelium (Supplementary Fig. 4). This observation was confirmed by a supervised analysis in which a total of 4421 genes were differentially expressed in fallopian tube tissue compared to the OSE and peritoneum, compared to 325 for ovarian tissue and 302 for peritoneal tissue (Supplementary Data 6). The 50 most differentially expressed genes from each tissue-specific analysis were combined into a signature of tissue type, which was used to characterize similarity between HGSC and normal tissue from each possible site of origin. Average Spearman correlation between tumor samples and fallopian tube tissue was 0.73, while the average correlation with ovarian tissue was 0.66 and the average correlation with peritoneal tissue was 0.56 (Fig. 7a). Significantly, 75/85 samples (88%, 95 percent CI = (79–94%)) were better correlated to fallopian tube tissue than to ovarian tissue, while peritoneal tissue provided the poorest match to all but one tumor (Fig. 7b).

## Discussion

For decades it was thought that ovarian carcinoma originates in the OSE or ovarian CICs^15^, but few data supported this claim^16–18^. Lesions resembling an intraepithelial carcinoma in CICs and the OSE are rare. In contrast, a putative precursor of ovarian carcinoma has been described in the fallopian tube, designated STIC, which is found frequently in association with “ovarian” HGSCs^4,19^ and in the fallopian tubes removed prophylactically from high-risk women^20^. These tubal lesions have a morphologic, immunohistochemical, and molecular genetic phenotype closely resembling ovarian HGSC.

Other molecular findings support the role of the distal fallopian tube in high-grade serous carcinogenesis. ALDH1A1 in human tissue samples has been demonstrated to be an early event in tumorigenesis being present in normal tubes cells, but lost in STIC lesions^21^. Additionally, RSF1 interacts with CCNE1 to promote tumorigenesis in a *TP53* mutant background^22^. Telomere shortening has also been found in STIC lesions supporting the role in early tumorigenesis^9^. Patterns of centrosome amplification in STIC lesions and HGSCs support the temporal relationship that STIC lesions precede the development of many HGSCs^23^. The distal fallopian tube and STIC lesions in particular are biologically plausible sites for early ovarian carcinogenesis and more likely than the long-held belief in the OSE.

Nonetheless, it is important to consider other proposed precursors (OSE and CICs) in order to reconcile the competing views of the origin of ovarian carcinoma. Though most data from model systems and human studies suggest that the distal fallopian tube is the site of origin for most high-grade serous ovarian cancers^3,24–30^, a minority of reports offers alternatives to this paradigm. A single case has been reported of a STIC lesion that was clonally related to a uterine endometrioid carcinoma^31^. CICs have been shown to contain aneuploidy cells and have increased proliferation compared to OSE, which may originate from invaginated fallopian tube epithelium during ovulatory repair^32^. Modification of a double knockout mouse model to include a p53 mutation in addition to previously reported Dicer-Pten suggests that these three alterations can induce HGSC to develop directly from the ovary and with the removal of Dicer, p53 and Pten can also cause this phenomenon^33^. When Pten is combined with Arid1a loss, mice develop endometrioid ovarian carcinoma rather than HGSC^34^.

We hypothesized that STIC is the precursor and not a metastasis of many, if not most, HGSCs. We have carefully characterized the morphologic and molecular genetic features of HGSCs and their associated precursor lesions. Molecular profiling has demonstrated the ability to distinguish tumors of different sites of origin. Gastrointestinal tumors originating from the appendix and the colon show similar morphologic features of mucinous differentiation, but have distinct gene expression profiles^35^. Gynecologic tumors of similar histologic subtypes but different organs or origin have also been shown to have different gene expression subtypes^36^. Additional examples exist for histologic subtypes of tumors that originate from similar anatomic sites such as breast and lung cancers that can be discriminated through both copy number alterations and gene expression^37–39^. If STIC is the precursor lesion to most HGSCs, the data generated by our studies will demonstrate molecular similarity between tumors with and without identifiable precursor lesions. If tumors with and without STIC lesions originate from independent sites of origin, we would expect multi-platform genomic analyses to display clear differences. Our data support the rationale to focus further efforts on the distal fallopian tube when considering surgical or medical approaches for the prevention of HGSC.

Analyses of the data from copy number alterations, messenger RNA sequencing and microRNA profiling fail to identify any significant differences between HGSCs with or without precursor STIC lesions. The few microRNAs that were differentially expressed between groups were not highly expressed in the data from TCGA, suggesting a limited role for these genes in ovarian cancer. If tumors with and without STIC lesions had different cellular origins, we would expect molecular distinctions to be identified based on this classification. In order to support our negative hypothesis, we performed various ancillary analyses to demonstrate the robustness of these data. We first confirmed that the focal copy number alterations identified in our data substantially overlapped with those identified by TCGA in their analysis of HGSC of presumed ovarian origin. We then confirmed the presence of four gene expression subtypes that have been reproducibly demonstrated in the published literature^12,14,40^. These findings highlight the consistent inter-patient heterogeneity seen in both this study population and the previously published TCGA work.

These data provide evidence from a multi-platform genomic perspective that HGSCs with or without STIC lesions originate from a common site of origin. However, one could argue that though the site of origin is likely to be similar between tumors with and without associated STIC lesions, from this data alone, it is difficult to conclude that the distal fallopian tube is that common site of origin. Therefore, to infer the most probable site of origin, we calculated the similarity between HGSC and normal fallopian tube, normal ovary and normal peritoneum through molecular barcodes, finding the most likely site of origin in the distal fallopian tube. This conclusion is supported by prior work, indicating that lineage markers, such as PAX8, for HGSC are shared with those from the distal fallopian tube and not the OSE or mesothelium^25,41^.

The implications of these findings are to direct efforts for prevention and early detection toward the distal fallopian tube, rather than the OSE. Ongoing and planned clinical trials are investigating bilateral salpingectomy with ovarian preservation as a viable option for ovarian cancer risk-reduction. Other promising approaches include direct sampling of normal fallopian tubes and detection of premalignant or early invasive tumors through shed biomarkers detected with pap smears or vaginal secretions^42,43^. These clinical trials and others will help to determine if the knowledge of ovarian cancer origins can be translated into broader and acceptable prevention approaches. Biomarker discovery will also be focused on the fallopian tube to identify better approaches for early detection and these data augment the scientific underpinning for reducing the burden of ovarian cancer.

## Methods

### Patients and tumor samples

We reviewed the records of all women diagnosed with HGSC of the pelvis, including diagnoses of ovarian, fallopian tube, or primary peritoneal carcinoma during the time period when our pathologists began routinely using SEE-FIM to evaluate the fallopian tubes. Patients with FIGO stage I to IV disease who had received primary cytoreductive surgery and pathologic processing of their fallopian tubes at one of the study sites were included. Patients with non-serous histologic subtypes (mucinous, endometrioid, clear cell) were excluded. Women with both optimal and suboptimal cytoreductive procedures were included. Women who had their primary surgery at an outside facility were excluded. Cases were all re-reviewed by a specialty gynecologic pathologist to confirm the presence or absence of STIC lesions using criteria published by the authors^44,45^. If sufficient tubal epithelium was not processed to determine whether or not a STIC lesion was present, the case was excluded from further consideration. Tumor specimens with <60% tumor cell nuclei were excluded. Qualifying cases were obtained from Memorial Sloan Kettering Cancer Center (*n* = 67, 79.8%), The Johns Hopkins Hospital (*n* = 13, 13.5%), and University Health Network (*n* = 16, 16.7%). Biospecimens were collected at diagnosis after obtaining informed consent. Local Institutional Review Board approval was obtained from each of the three contributing sites. Normal epithelial cells from ovarian, fallopian tube and peritoneal tissue were collected through cytologic brushings to provide a normal gene expression profile from each tissue type. These brushings were collected in the operating room from patients without cancer during surgical procedures upon entry into the abdomen and prior to vascular ligation or other intraoperative manipulation. Representative photomicrographs confirmed that the target normal tissue type was collected without substantial contamination from adjacent tissues (i.e., fallopian tube and ovarian surface brushings were estimated to contain <10% of cells from the non-targeted tissue site).

### Nucleic acid isolation

DNA and RNA were co-isolated from frozen tissue tumor blocks, and RNA was isolated from normal tissues utilizing Ambion’s ToTALLY RNA™ RNA Isolation Kit (Part Number AM1910, ©Ambion, Inc.). This protocol allows sequential isolation and collection of both DNA and RNA. The concentration of DNA and RNA was initially obtained using a NanoDrop spectrophotometer in our laboratory. DNA concentration was confirmed using a PicoGreen protocol. RNA quality was confirmed by utilizing an Agilent Bioanalyzer and assigning an RNA integrity number (RIN) to each sample.

### Somatic copy number data

Total genomic DNA (500 ng) was collected from all 96 samples included in the study, and digested with Nsp I and Sty I restriction enzymes and ligated to adapters. PCR conditions preferentially amplify fragments in the 200–1100 bp size range. PCR amplification products for each restriction enzyme digest are combined and purified using polystyrene beads. The amplified DNA is then fragmented, labeled, and hybridized to a SNP Array 6.0 containing > 906,600 single-nucleotide polymorphisms (SNPs) and > 946,000 probes for the detection of copy number variation. All 96 samples were assayed at once, in a single batch.

### MicroRNA expression data

Total RNA (100 ng) was obtained from 95 of 96 samples, and hybridized to the nCounter Human miRNA sample probes (Seattle, WA). Subsequently, the samples were placed into the nCounter Prep Station for automated sample purification and subsequent reporter capture. Each sample was scanned for 600 FOV on the nCounter Digital Analyzer to extract the data. All 95 samples were assayed in a single batch.

### RNA sequencing library preparation

RNA sequencing libraries were prepared using the Illumina TruSeq Stranded Total RNA Sample Preparation Kit. Briefly, 500 ng of total RNA was purified by Ribo-Zero to remove rRNA and fragmented by divalent cations under elevated temperature. The fragmented RNA underwent first strand synthesis using reverse transcriptase and random primers. Second strand synthesis created the cDNA fragments using DNA polymerase I and RNaseH. The cDNA fragments then went through end repair, adenylation of the 3′ ends, and ligation of adapters. The cDNA library was enriched using 10 cycles of PCR and purified. Quality control consisted of assaying the final library size using the Agilent Bioanalyzer and quantifying the final library by real-time PCR and PicoGreen (fluorescence) methods. A single peak between 250 and 350 bp indicates a properly constructed and amplified library ready for sequencing. Eleven of 96 samples failed RNA quality criteria, or library prep and the remaining 85 samples were analyzed in two batches, with STIC and NOSTIC samples well-represented in both.

### RNA sequencing

Sequencing was performed on the HiSeq 2500 instrument using v4 SBS chemistry according to the Illumina protocol, as described in Bentley et al^46^. Sequencing libraries were loaded onto the HiSeq 2500 flowcell for clustering on the cBot using the instrument specific clustering protocol. The HiSeq 2500 is capable of generating 200–250 M passed filter 2 × 50 bp sequencing reads per flow cell lane. In order to obtain a minimum of 40 M PF reads per sample, we sequenced 6 samples per lane.

### Bioinformatics analysis

RNA sequences were aligned to the NCBI human genome build 37 using STAR aligner (v2.3.1z)^47^. Quantification of genes annotated in Gencode (version 18) was performed using featureCounts (v1.4.3)^48^. DESeq2 (doi:10.1101/002832) was used to normalize feature counts. Picard and RSeQC^49^ was used to collect QC metrics (http://broadinstitute.github.io/picard/).

### Molecular analyses

Post-alignment normalization of the RNAseq data and differential expression analysis was performed using Voom in the limma package from Bioconductor^50–52^. The nanoStringNorm procedure as implemented in the nanoStringNorm Bioconductor package was used to preprocess the miRNA data, followed by differential expression analysis with Empirical Bayes methods via the limma Bioconductor package^52,53^. miR pathway analysis was performed with Diana Tools miRPath v2.0^54^.

The Affy Power Tools (APT) copy number workflow was used to preprocess the SNP arrays and derive log R ratios representing relative copy number abundance^55^. A standard HapMap reference included in the APT toolbox was used to represent a diploid genome. Circular binary segmentation as implemented in the DNAcopy (Seshan VE and Olshen A. DNAcopy: DNA copy number data analysis. R package version 1.42.0) package from Bioconductor, using standard settings^51,56^. The GISTIC2.0 algorithm was applied to the segmented data via the genome pattern online tool suite, using default settings, to make gene level copy number calls and identify significantly, copy number altered regions^57^.

### Comparative analysis

Statistical analyses were performed and figures prepared in the R statistical software suite using standard functions and custom routines^58^. Unsupervised cluster analyses were performed using the hclust function with Euclidean and Pearson Correlation distances and Ward’s method. Analyses of overall and progression-free survival were performed under Cox proportional hazards assumption, with a *P* value of 0.05 considered statistically significant. Differentially expressed genes were considered statistically significant at a Benjamini–Hochberg FDR of 10%^59^. A *t*-test was used to test for association between STIC status and age, and Fisher’s exact test was used to evaluate potential associations between STIC status and clinical covariates including race, stage, platinum response, and surgical outcome.

### Subtyping tumors

The TCGA network identified 4 subtypes of ovarian cancer based on RNA expression patterns^13^, together with a set of 100 genes selected to optimally classify HGSC tumors. We defined a prototype expression profile for each subtype by calculating the average expression for each gene, over samples assigned to the subtype. Then, for each tumor sample, we calculated the correlation with each subtype profile, definitively assigning the tumor to the best-correlated class only when there was a single, clear best match. Specifically, a definitive assignment was made only if the maximum correlation was at least 1.5 × the second highest value.

Tissue-specific expression patterns were identified by performing differential expression analysis comparing each tissue type to the other two^52,53^. The 50 most differentially expressed genes from each tissue-specific analysis were combined into a signature of tissue type. Tumors were matched to normal expression profiles obtained from normal ovarian, fallopian tube and peritoneal tissue, by correlation as described above for TCGA expression subtypes. Specifically, correlation coefficients were calculated between each tumor and each normal pool, over the tissue-specific genes. Each tumor was assigned to the tissue type with the highest average correlation.

### Power analyses

Our key assumption was that distinct cancer types would show moderate differences (0.75–1.25 s.d.) in expression in many genes and large differences (>1.5 s.d) in some genes, with similar differences in other data types. Accordingly, we developed a study with sufficient power to detect most differences in this range, reasoning that a negative result would provide strong evidence in support of the single origin theory. We calculated power by simulation, assuming that t-tests are used to detect differential expression, with type-I error controlled at an FDR of 0.10.

Assuming that even 50 out of 20,000 genes were differentially expressed, with 40–50 samples per group, we expect to detect many differentially expressed genes at an effect size of 0.75 s.d., most genes at an effect size of 1.0 s.d., and virtually all with an effect size of 1.5 s.d. or above, as shown below. Detection rates are higher when more genes are differentially expressed (Supplementary Table 1). Our final sample accrual of 48 samples per group fell well within these bounds. SNP arrays were completed for all 96 samples, miRNA analysis was assessed by nanostring in a total of 95 samples (48 STIC, 47 NOSTIC), and RNAseq was performed on 85 samples, (43 STIC, 41 NOSTIC).

### Data availability statement

All the genomic data including microRNA, RNAseq and copy number data have been deposited in the GEO database under the accession code GSE102094. The TCGA data referenced in this study are available in a public repository from the National Cancer Institute’s Genomic Data Commons at https://gdc.cancer.gov/. The authors declare that all the other data supporting the findings of this study are available within the article and its supplementary information files and from the corresponding author upon reasonable request.

## Electronic supplementary material

Supplementary Information

Description of Additional Supplementary Files

Supplementary Data 1

Supplementary Data 2

Supplementary Data 3

Supplementary Data 4

Supplementary Data 5

Supplementary Data 6
