# Supplementary material for: Molecular analysis of high-grade serous ovarian carcinoma with and without associated serous tubal intra-epithelial carcinoma
Source: Nat Commun. 2017 Oct 17;8:990. doi: 10.1038/s41467-017-01217-9 (PMC5645359; doi:10.1038/s41467-017-01217-9)
Supplement: Supplementary file 1 — Supplementary Information [file 41467_2017_1217_MOESM1_ESM.pdf]

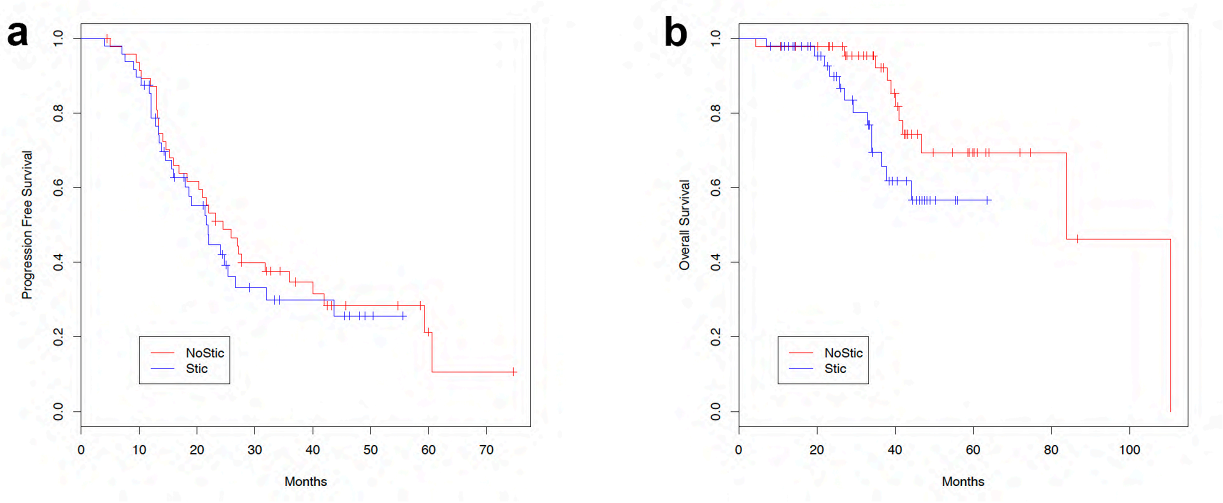

Supplementary Figure 1. Clinical outcome by STIC status. Progression-free (a; p = 0.56, log-rank test) or overall (b; p=0.084, log-rank test) survival are shown for patients with or without STIC lesions.

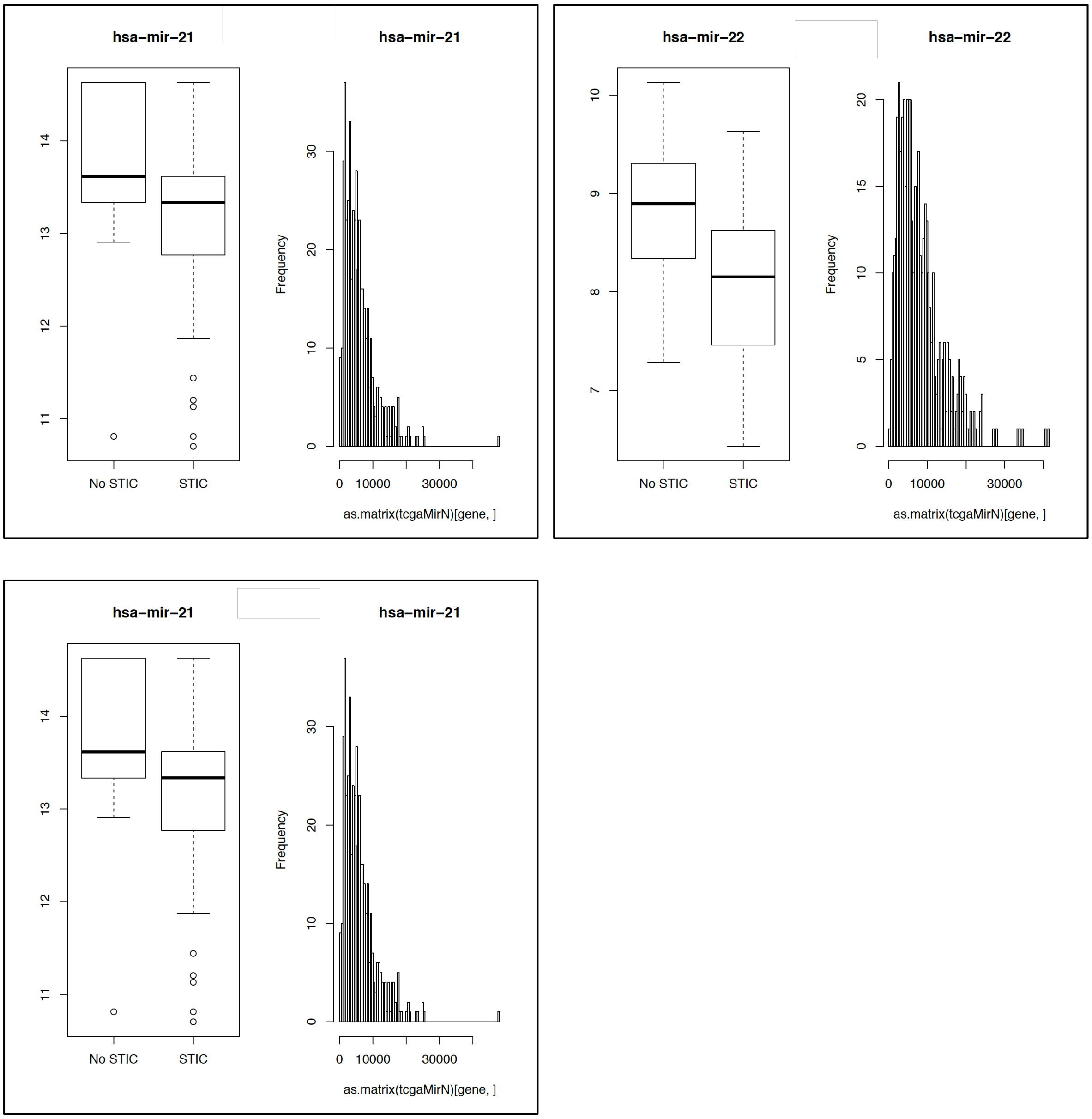

Supplementary Figure 2. Expression of microRNAs in this study and TCGA. Each left panel shows the expression of a given microRNA in the current study and in TCGA (right panel). Boxplot center lines represent medians, box limits are the inter-quartile range from 25% and 75%, whiskers represent the extent of tumors out to 1.5 times the inter-quartile range, and circles are outliers beyond 1.5 times the inter-quartile range.

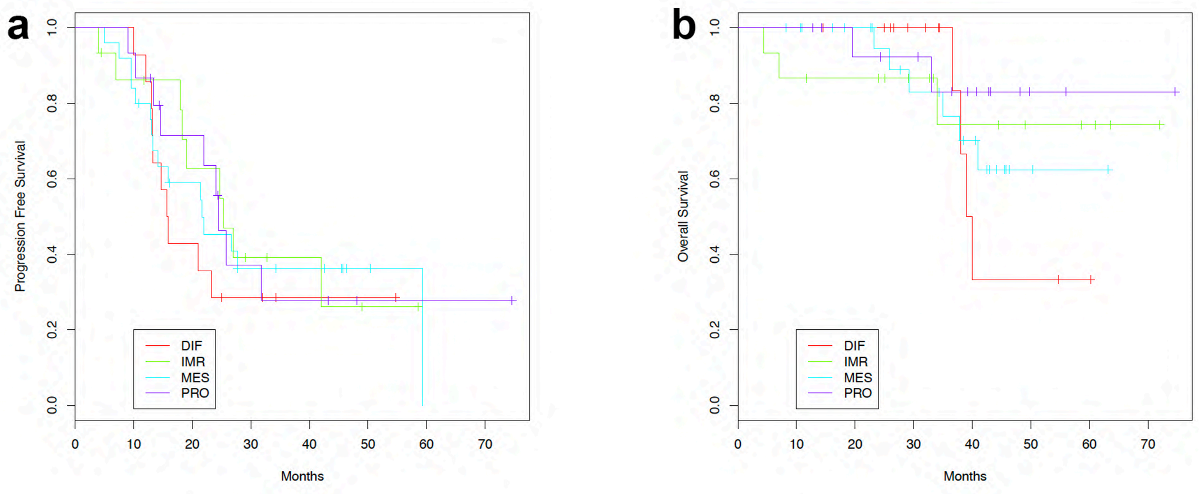

Supplementary Figure 3. Clinical outcome by gene expression subtype. Progression-free (a; p = 0.81, log-rank test) or overall (b; p=0.71, log-rank test) survival between gene expression subtypes.

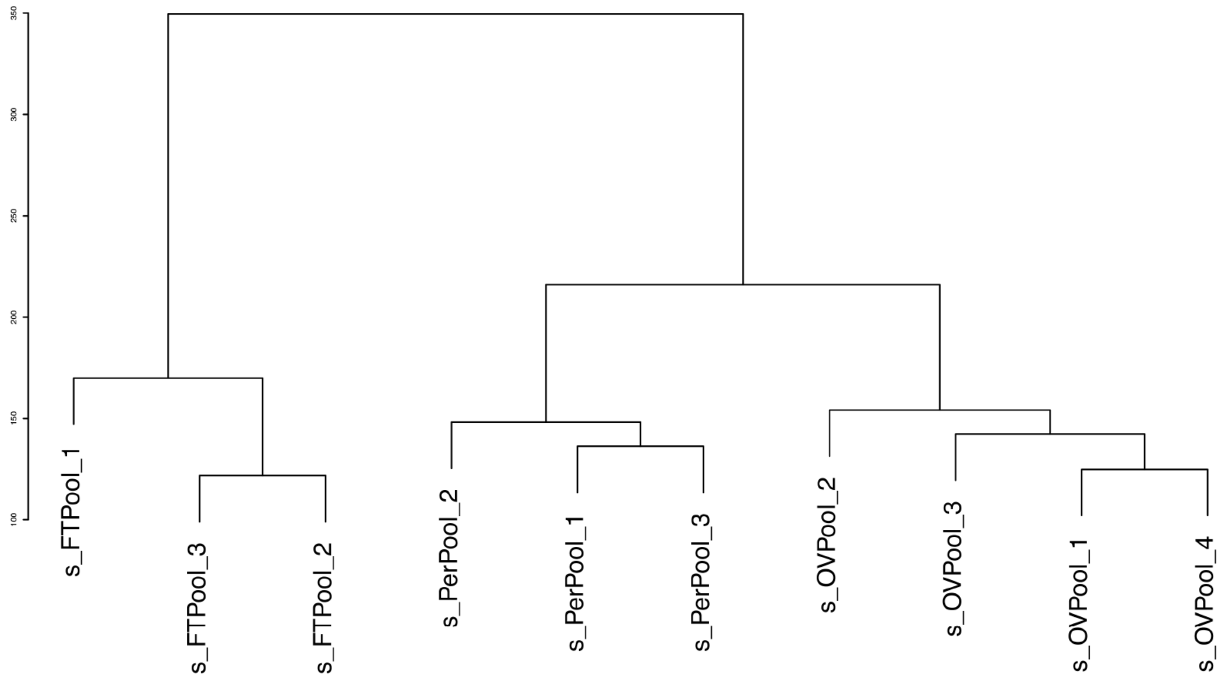

Supplementary Figure 4. RNA sequence clustering of normal tissues. Unsupervised hierarchical clustering of RNA sequence data from pooled tissue samples of ovarian surface epithelium (OV), peritoneum (Per), and fallopian tube epithelium (FT).

Power for Molecular Studies		
Effect Size	Sample Size<br>Reported as percent detected, with 90% C.I.	
	40 per group	50 per group
0.75s.d.	20% (CI: 8% to 34%)	40% (90% CI: 24% to 54%)
1.0s.d.	71% (58%-82%)	88% (80%-94%)
1.5s.d.	99% (98%-100%)	99.99% (>99%)

Supplementary Table 1. Power analysis framework for molecular studies.
