# Supplementary material for: Molecular analysis of high-grade serous ovarian carcinoma with and without associated serous tubal intra-epithelial carcinoma
Source: Nat Commun. 2017 Oct 17;8:990. doi: 10.1038/s41467-017-01217-9 (PMC5645359; doi:10.1038/s41467-017-01217-9)
Supplement: Supplementary file 2 — Description of Additional Supplementary Files [file 41467_2017_1217_MOESM2_ESM.pdf]

Description of Additional Supplementary Files

File Name: Supplementary Data 1
Description: Detailed demographic, molecular and clinical characteristics of individual patients with high-grade serous carcinomas with and without STIC lesions.

File Name: Supplementary Data 2.
Description: Copy number alterations identified as significantly altered in the GISTIC analysis compared between samples with and without STIC lesions by genomic region.

File Name: Supplementary Data 3.
Description: Copy number alterations identified as significantly altered in the GISTIC analysis compared between samples with and without STIC lesions by gene.

File Name: Supplementary Data 4.
Description: Class comparison of mRNA expression between samples with and without STIC lesions.

File Name: Supplementary Data 5.
Description: Class comparison of microRNA expression between samples with and without STIC lesions.

File Name: Supplementary Data 6.
Description: Differentially expressed genes among normal tissue subtypes.
